# Ulinastatin ameliorates podocyte ferroptosis via regulating miR-144-3p/SLC7A11 axis in acute kidney injury

**DOI:** 10.1007/s11626-023-00814-x

**Published:** 2023-10-11

**Authors:** Xiaosong Yang, Na Guo

**Affiliations:** 1 Department of Anesthesiology, State Key Laboratory of Oncology in South China, Guangdong Provincial Clinical Research Center for Cancer, Sun Yat-Sen University Cancer Center, Guangzhou, China; 2 Guangdong Esophageal Cancer Institute, Guangzhou, China

**Keywords:** Ulinastatin (UTI), Ferroptosis, Acute kidney injury (AKI), Solute carrier family 7 member 11 (SLC7A11)

## Abstract

Ferroptosis is a newly discovered form of cell death characterized by intracellular iron accumulation and subsequent lipid peroxidation, which has been identified in various pathological processes, such as acute kidney injury (AKI). Ulinastatin (UTI), known as an antioxidant and anti-inflammatory, has been reported to prevent kidney injury. Here, we investigated the protective effects of UTI on LPS-induced podocyte ferroptosis in vivo and in vitro. Conditionally immortalized mouse podocyte was exposed to LPS in the presence or absence of UTI in vitro for 48 h. The levels of reactive oxygen species (ROS) and intracellular Fe^2+^ were detected to value the effect of UTI treatment on the podocyte cell ferroptosis. We also evaluated the influence of UTI on kidney injury in vivo. LPS-induced mice were treated with vehicle or UTI at 50 U/g/d for 6 wk. We identified the important function of UTI in repressing ferroptosis and ameliorating podocyte injury. The treatment of UTI reduced accumulation of Fe^2+^ and lipid ROS in podocyte. The cell proliferation was induced by UTI compared with the LPS-treated group in vitro. UTI attenuated the podocyte cytoskeletal as well. Regarding the mechanism, we found that UTI upregulated solute carrier family 7 member 11 (SLC7A11) expression by reducing miR-144-3p in the cells. The overexpression of miR-144-3p blocked the protective role of UTI in podocyte ferroptosis. MiR-144-3p/SLC7A11 axis was involved in UTI-mediated podocyte cell proliferation in vitro. Furthermore, the treatment of UTI repressed podocyte injury and proteinuria in vivo, and the level of miR-144-3p was decreased while SLC7A11 expression was increased in comparison with the model mice. UTI prevents LPS-induced podocyte ferroptosis and subsequent renal dysfunction through miR-144-3p/SLC7A11 axis. These findings might provide a potential novel therapeutic option for AKI and other renal diseases affecting podocyte.

## Introduction

Kidney injury can be divided into acute kidney injury (AKI) and chronic kidney injury (CKD) according to the disease development progression, and it is a common condition associated with morbidity and mortality of patients (Wei et al. 2012). Kidney injury continues to be an ongoing problem, with an overall incidence of AKI of 33%, and a 5% incidence of renal injury that requires dialysis (Guo et al. 2019). AKI is common, dangerous, and costly, which is a serious problem in hospitalized patients. Sepsis is the leading cause of acute kidney injury in critically ill patients (Poston and Koyner 2019). AKI represents an acute decline in renal function that leads to structural changes. Glomerular disease is a related cause of AKI, characterized by a reduction of glomerular filtration rate or urine output (Fenoglio et al. 2019). Podocytes are terminally differentiated epithelial cells that reside on the glomerular basement membrane outside the glomerular capillaries (Schwartzman et al. 2016). They are key components of the ultrafiltration system in glomeruli. Podocyte foot processes interdigitate with the counterparts of their neighboring cells to form filtration slits, known as the slit diaphragm (Nagata 2016). Several proteins are implicated in maintaining podocyte structural organization, including synaptopodin and nephrin. Podocytes are highly susceptible to damage during kidney injury, and genetic defects to podocytes frequently result in severe kidney disease (Guo et al. 2016). Podocyte injury and apoptosis have been reported to be a pivotal factor in glomerular diseases and, in particular, different causes of AKI, such as renal ischemia–reperfusion (I/R) injury and nephrotoxicity, have been shown to be related closely to podocyte injury (Kang et al. 2010).

Ferroptosis, a type of iron-dependent regulated cell death mediated by lethal accumulation of lipid peroxides, has attracted much attention recently (Dixon et al. 2012). Ferroptosis is implicated in numerous human diseases and pathologies, including neurodegenerative diseases and cancers (Hu et al. 2019; Wu et al. 2020; Yee et al. 2020). Studies have shown that ferroptosis occurred after AKI and the intervention of ferrostatin-1 (Fer-1), a ferroptosis inhibitor, has a renoprotective effect in I/R kidney injury (Hu et al. 2019). Solute carrier family 7 member 11 (SLC7A11, also called xCT), the catalytic subunit of the cystine/glutamate antiporter system Xc(−), is the major transporter of extracellular cystine. Moreover, exploring the regulatory mechanisms of SLC7A11 has been a focus with significant importance for its role in maintaining intracellular glutathione levels and protecting cells from oxidative-stress–induced cell death, and targeting SLC7A11 has been implicated in multiple studies (Zhang et al. 2019). Studies in recent years have linked SLC7A11 to ferroptosis in various cancers and heart disease (Koppula et al. 2021).

Ulinastatin (UTI), a multivalent enzyme inhibitor, was first identified and purified from human urine (Liu et al. 2017). Because of its excellent anti-inflammatory and antioxidant effects, UTI is widely used in the treatments of pancreatitis, sepsis, and sepsis-related multiple-organ dysfunction, reducing the mortality effectively. Experimental studies have shown organ-protective effects of UTI on I/R injury of the lung, liver, heart, and kidney (Wan et al. 2016). UTI attenuates LPS-induced inflammation and inhibits endoplasmic reticulum stress-induced apoptosis in renal tubular epithelial cells via regulation of the TLR4/NF-κB and Nrf2/HO-1 pathways. Interestingly, Wang and his colleagues have revealed that UTI protects against acetaminophen-induced liver injury by alleviating ferroptosis via the SIRT1/Nrf2/HO-1 pathway (Wang et al. 2021).

MicroRNAs (miRNA or miR) are 20–24-nt-long non-coding RNAs, which fine-tune the expression of the majority of human genes by inhibiting translation and accelerating mRNA decay (Trionfini and Benigni 2017). miRNAs have been associated with the protective effects of UTI in lung and peripheral nerve injury. On the other hand, previous studies verified miR-144-3p was involved in kidney injury by regulating cell apoptosis and oxidative stress (Lin et al. 2014). Additionally, ferroptosis can be controlled by epigenetic regulation. Previous studies have reported the importance of miRNA in regulating ferroptosis. AKI is histologically characterized by necrotic cell death and inflammation. Thus, we hypothesized that UTI could alleviate ferroptotic podocyte in AKI through downregulating miR-144-3p. The present study attempted to explore the potential regulatory mechanism of UTI on ferroptosis *in vivo* and *in vitro*.

## Materials and methods

### Cell culture and treatment

The conditionally immortalized mouse podocyte cell line (MPC5) was kindly provided by Prof. Jochen Reiser (Rush University Medical Center, Chicago, IL). The cells were taken out of the refrigerator, resuscitated, and cultured in RPMI-1640 culture medium containing 10% fetal bovine serum with interferon-γ (50 U/mL), conducting proliferation in 5% CO_2_ incubator at 33 °C. Observation the growth state of cells, and when the cells are fused to 85%, the culture medium without interferon-γ was changed and cells were incubated in 5% CO_2_ incubator at 37 °C for 14 d.

The culture medium was changed 24 h before the experiment. We collect MPC5 cells in good growth condition, adjust the density, and inoculate them in a 96-well plate. The MPC5 cells were labeled as control group and LPS (1 μg/mL) treated cells were recorded as LPS group; the LPS group treated with UTI was recorded as the UTI group. The ferroptosis markers and miR-144-3p expression were detected. For the other part of the study, the cells treated with UTI and LPS were recorded as the UTI group; the miR-NC and miR-144-3p mimics were transfected into MPC5 cells according to the Lipofectamine™ 2000 kit, and then treated with UTI and LPS, which were recorded as miR-NC and miR-144-3p groups, and then SLC7A11 expression levels were detected.

### Animal studies

Male BALB/c mice (weighing about 20–22 g) were purchased from the experimental animal center of Sun Yat-sen University (Guangzhou, China). BALB/c mice were randomly divided into 3 groups (*n* = 6/each group): (1) Normal BALB/c mice were chosen as the control group; (2) BALB/c mice were given a single intravenous injection of LPS (10 mg/kg, intraperitoneal injection) to establish a model of LPS nephropathy (LPS group); (3) LPS-induced mice treated with UTI at 50 U/g/d for the next 6 wk (UTI group). The control and LPS groups were treated with the equivalent volume of normal saline within the same time. At week 6, each mouse was placed in a metabolic cage to collect 24-h urine, respectively. Urine samples were centrifuged at 3000 × *g* for 10 min at 4 °C to remove particulate contaminants and stored at − 80 °C. After the urine was collected, the mice were euthanized, and the blood and kidney tissues (isolated glomeruli) were collected for subsequent analysis. All experiments involving animals were approved by the Animal Care and Use Committee of Sun Yat-sen University Cancer Center. All mice were treated with humane care according to the criteria outlined in the “Guide for the Care and Use of Laboratory Animals.”

### Cell proliferation

MPC5 cells of each group were collected. After trypsin digestion and culture, the cells were inoculated into 96-well cell plates for 48 h. Add 150 μL DMSO solution to the supernatant of cells after centrifugation, detect the OD value of each group of cells at 540-nm wavelength of the microplate reader, and calculate the cell activity.

### Histomorphometry and immunofluorescence

Periodic acid-Schiff (PAS) staining steps are as follows: renal tissue is sliced; dewaxed with xylene; washed with ethanol; then hydrated with 95%, 85%, and 75% ethanol respectively; stained with Weigert iron hematoxylin solution for 5 min. Then the slide was washed with water, stained with alicin blue solution for 10 to 20 min, put in an oxygenator for oxidation for 5 min, impregnation in Schiff Agent for 10 min, and stained with hematoxylin solution for 1 to 2 min. Then Scott blue solution was used to turn blue. After that, we perform dehydration, transparency, and film sealing treatment, and then perform microscopic examination. The results of PAS staining were statistically analyzed with ImagePro Plus 6.0 system, and the index was calculated. Each group of pictures was repeated 3 times.

The cells were washed with PBS three times, fixed at room temperature with 4% paraformaldehyde for 15 min, and washed with PBS for three times. The membrane was broken at room temperature with 0.3% Triton X-100 for 10 min and washed with PBS for three times. The goat serum was sealed at room temperature for 1 h, and the primary antibody was added. The goat serum was shaken at 4 °C overnight, washed with PBS three times, added with fluorescent secondary antibody (1:500) and incubated at room temperature for 1 h in the dark, added with DAPI for 5 min, and then sealed with an anti-fluorescence quenching agent, observed, and photographed under the immunofluorescence microscope. Fluorescence intensity was analyzed by ImagePro Plus image analysis software. Rhodamine phalloidin (Cytoskeleton Inc., Denver, CO) staining was used to show actin rearrangement as indicated by the manufacturer’s instructions.

### Glomerulosclerosis index

Glomerulosclerosis was defined as basement membrane thickening, mesangial hypertrophy, and capillary occlusion. The glomerulosclerosis index (GSI) was assessed in PAS-stained sections in 80 randomly selected glomeruli, and the degree of sclerosis was graded using a semiquantitative scoring method, as previously described (Advani et al. 2011): grade 0, normal; grade 1, sclerotic area up to 25% (minimal); grade 2, sclerotic area 25 to 50% (moderate); grade 3, sclerotic area 50 to 75% (moderate to severe); and grade 4, sclerotic area 75 to 100% (severe).

### Electron microscopy

After the experiment, kidney tissue was fixed with 2.5% glutaraldehyde and 0.22 mmol/L phosphate buffer solution for about 4 h, then rinse it with 0.1 mol/L phosphoric acid for 10–15 min, repeat it three times, fix it with 1% osmic acid for 2 h, dehydrate it with ethanol step by step, embed it with epoxy resin passing the night at 30 °C, bake it in a 60 °C oven for 12 h, and cut it into thin slices with a thickness of 50–100 nm. Finally, double staining with uranium acetate and lead nitrate was performed under a transmission electron microscope.

### Iron assay

Take MPC5 cells with good growth in logarithmic phase, treated as mentioned above. The relative cellular ferrous iron (Fe^2+^) level in the cell lysates was checked with an iron assay kit (Abcam, ab83366) according to the manufacturer’s instructions. Briefly, after treatments, MPC5 were collected and washed in cold PBS three times. Then, 200 µL iron assay buffer was added to each well of the cells on ice. Cell lysates were collected, and an iron reducer was added to reduce the switch from Fe^3+^ to Fe^2+^, mixed carefully, and reacted for 30 min. Subsequently, the iron probe was added and mixed thoroughly and then incubated for 60 min. The output was measured immediately at 593 nm by a spectrophotometer (BioTek-Epoch2).

### Lipid ROS assays

MPC5 cells treated with C11-BODIPY fluorescent probe (10 μmol/L) were incubated for 30 min; the cells were harvested in 15-mL tubes and washed with PBS twice. Then the cells were resuspended in 500 µL PBS. The cell suspension was filtered through a cell strainer (40-µm nylon mesh) and subjected to flow cytometric analysis to examine the amount of ROS within the cells. The fluorescence intensities of the cells per sample were determined by flow cytometry using a BD FACSAria flow cytometer (BD Biosciences, San Jose, CA).

### Dual-luciferase activity assay

Cells were divided into miR-144-3p mimics and miR-144-3p NC (negative control). The cell culture medium was moistened twice using PBS solution and then cell lysate was added. The sample, firefly luciferase test reagent, and *Renilla luciferase* test reagent were balanced to room temperature, and then perform the following operations: add 100 μL firefly luciferase detection reagent to each hole, add 20 μL sample, gently blow and mix with a pipette, and then read. The cell lysate was used as blank control; add 100 μL *Renilla luciferase* detection reagent per hole, use the microplate reader to vibrate and mix well, and then read. With *Renilla luciferase* as the internal reference, divide the relative light unit (RLU) value measured by firefly luciferase by the RLU value measured by *Renilla luciferase*. According to the ratio obtained, the activation degree of the target reporter gene among different samples was compared.

### RNA extraction and real-time PCR

Collect MPC5 cells of each group and extract the total RNA. In total, 5 μg RNA was reverse-transcribed into cDNA according to the instructions of the reverse transcription kit, and the reaction system was configured according to the fluorescent quantitative kit for PCR amplification. Using U6 as an internal parameter, the expression of miR-144-3p was calculated by the 2^−△△Ct^ method.

### Western blot analysis

Collect MPC5 cells of each group and add RIPA lysate to lyse them on ice for 5 min. The supernatant was collected by centrifugation with 12,000 × *g*. The protein was transferred to PVDF membrane after SDS-PAGE gel treatment, and blocked for 1.5 h by adding 50 g/L nonfat milk. Desmin (1:1000), synaptopodin (1:200), SLC7A11 (1:1000), and β-actin antibody (1:10,000) were incubated at 4 °C. The next day, HRP labeled goat anti rabbit IgG was added and incubated at room temperature for 1.5 h. Electrochemiluminescence solution was dropped in the dark room for development. Read the gray value of protein stripe through ImageJ software to β-actin (used as internal reference) to analyze the expression of target protein.

### Statistical analysis

All statistical analyses were conducted using SPSS 17.0 and R (version 4.3.1). The Shapiro–Wilk test was used to test normality and Bartlett’s statistic was used for homogeneity of variances. Student’s *t*-test (two groups) or ANOVA (more than two groups) was used to analyze the differences among various groups. Furthermore, the Tukey honest significant difference (HSD) test was used to compare the differences between groups, and *P* < 0.05 was considered statistically significant.

## Results

### UTI inhibits LPS-induced podocyte ferroptosis

**Figure 1.**
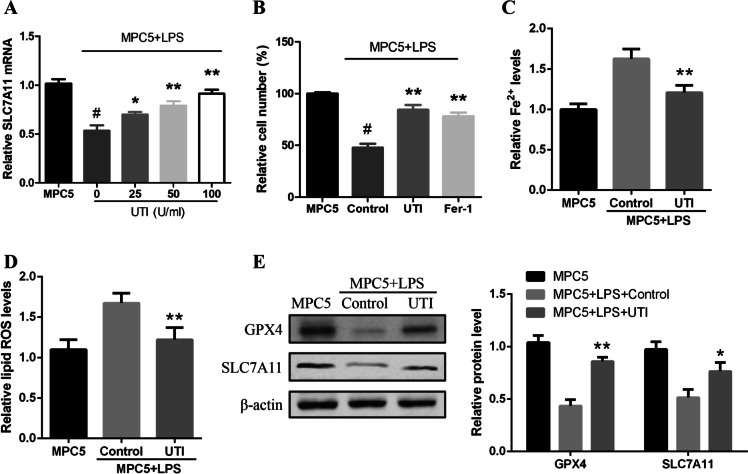
UTI inhibits LPS-induced podocyte ferroptosis. (*A*) MPC5 cells were treated with UTI at the indicated concentrations. The mRNA expression of SLC7A11 was analyzed by qPCR. (*B*) MPC5 cells were treated with UTI (100 U/mL) and Fer-1 (5 mmol/L). The cell viability was detected by MTT assays after 48 h of the treatment. MPC5 cells were treated with UTI (100 U/mL). The Fe^2+^ (*C*) and lipid ROS levels (*D*) were detected. (*E*) The expression of GPX4 and SLC7A11 was measured by western blot analysis. The experiments were performed independently three times. *P < 0.05 and **P < 0.01 versus LPS group; ^#^*P* < 0.05 versus MPC5 group.

We were interested in the function of UTI in regulating LPS-induced podocyte ferroptosis. Our data showed that UTI upregulated the mRNA expression of SLC7A11 in a dose-dependent manner in LPS-treated podocyte at 48 h, in which 100 U/mL UTI presented the highest effect and was selected in the subsequent analysis (Fig. 1*A*). The treatment of UTI was able to inhibit of cell death (Fig. 1*B*). Ferrostatin-1 (Fer-1), a ferroptosis-specific inhibitor, also alleviates cell death, suggesting that ferroptosis plays an important role in LPS-induced podocyte injury (Fig. 1*B*). Meanwhile, ferrous iron (Fe^2+^) was analyzed in the cells. The levels of Fe^2+^ were induced by LPS and ameliorated by UTI in MPC5 cells (Fig. 1*C*). In addition, LPS promoted lipid ROS accumulation in podocyte cells which was reduced by UTI treatment (Fig. 1*D*). The expression of SLC7A11 and GPX4 was raised by UTI in MPC5 cells (Fig. 1*E*). Taken together, these data suggest that UTI inhibits LPS-induced podocyte ferroptosis.

### UTI ameliorated LPS-induced podocyte injury

**Figure 2.**
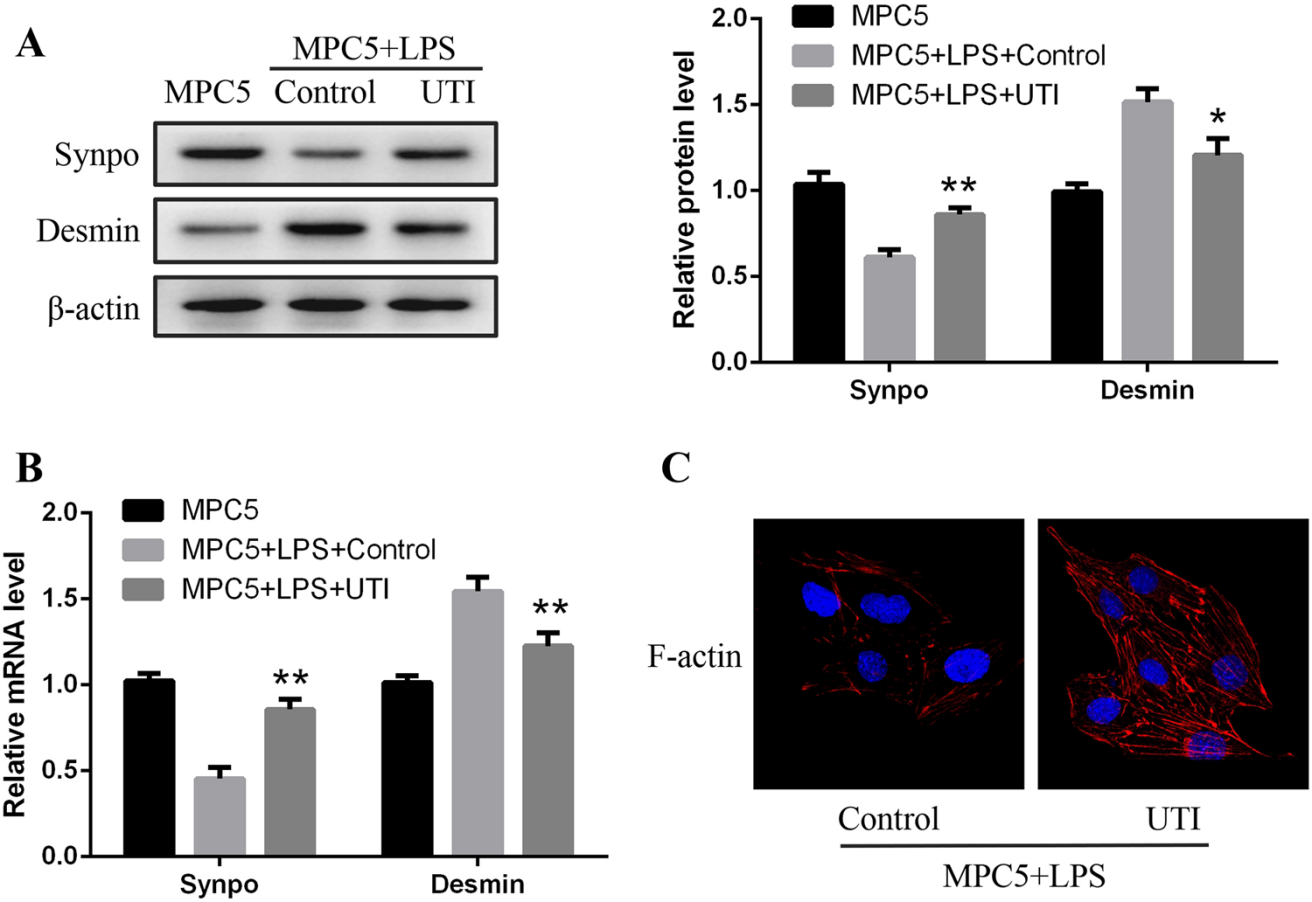
UTI ameliorated LPS-induced podocyte injury. (*A*) Western blotting and (*B*) qRT-PCR showing the protein and mRNA levels of synaptopodin and desmin in MPC5, LPS, and LPS + UTI groups. (*C*) Effects of UTI on cytoskeletal stabilization in podocytes (original magnification × 120). *P < 0.05 and **P < 0.01 versus LPS group.

Synaptopodin is implicated in maintaining podocyte structural organization, while desmin is a biomarker of podocyte injury. The research of Herrmann A found that, in the model for acute podocytes injury, desmin staining was enhanced in podocytes (Herrmann et al. 2012). Based on these indicators, we then observed that the LPS-induced MPC5 cell injury was alleviated by UTI significantly. It was shown that the protein and mRNA levels of synaptopodin were decreased in the LPS group; meanwhile, desmin expression was increased compared with the normal MPC5 group. Treatment with 100 U/mL UTI significantly protected against LPS-induced podocyte injury (Fig. 2*A*, *B*). After treatment with LPS, the podocyte cytoskeleton was in disorder and rearranged which may result in podocyte dysfunction and injury, while UTI promoted the rearrangement of stress fibers in podocytes, causing them to localize around the cytomembrane (Fig. 2*C*).

### UTI upregulated SLC7A11 expression by inhibiting miR-144-3p

**Figure 3.**
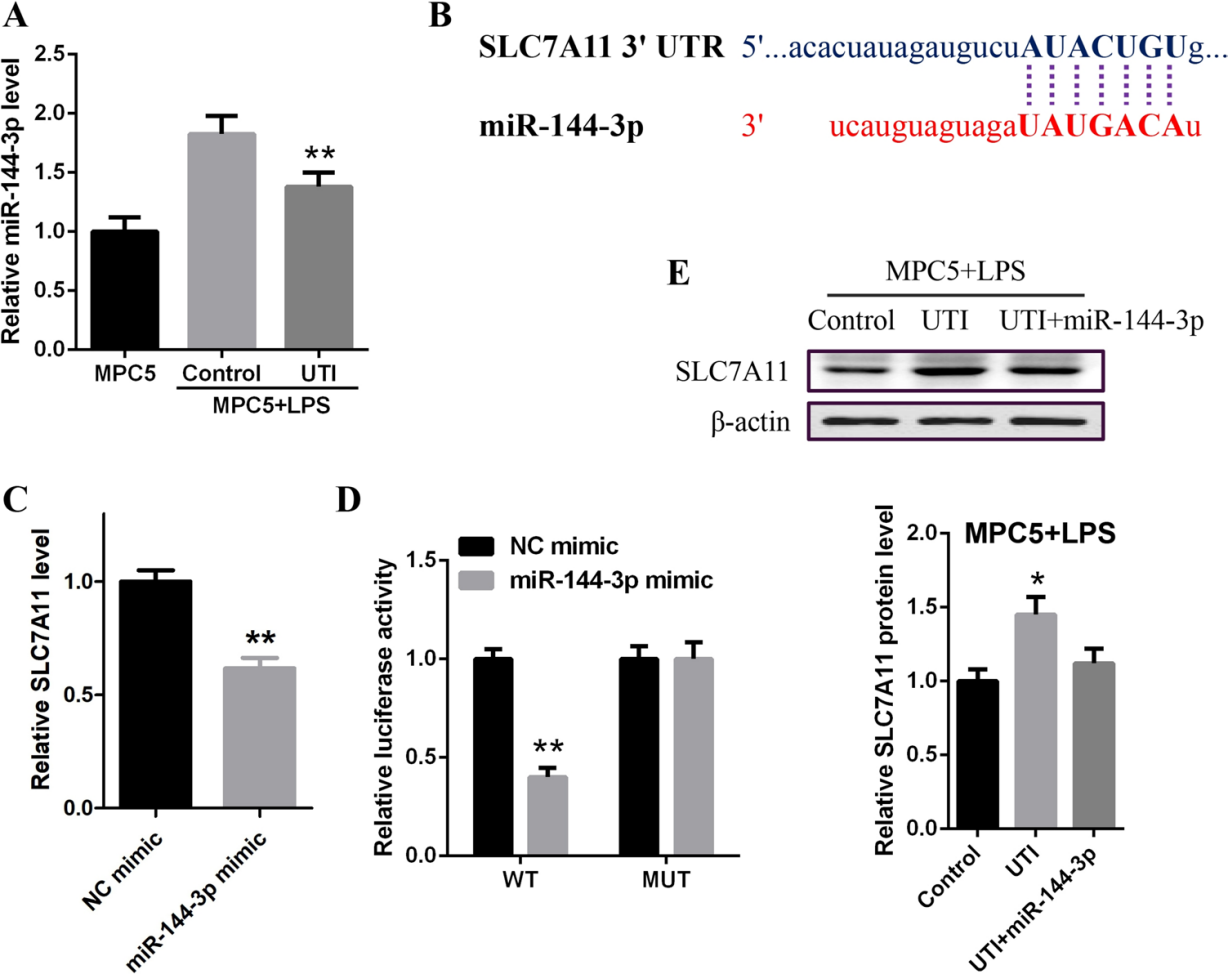
UTI upregulated SLC7A11 expression by inhibiting miR-144-3p. (*A*) MPC5 cells were treated with UTI (100 U/mL). The expression of miR-144-3p was analyzed by qPCR assays. (*B*) We predicted the binding site of miR-144-3p and SLC7A11 in a bioinformatic analysis. (*C*) MPC5 cells were treated with miR-144-3p mimic. The mRNA levels of SLC7A11 were examined by qPCR. (*D*) The luciferase activity of SLC7A11 mRNA 3′-UTR was analyzed. (*E*) The protein expression of SLC7A11 was detected by western blot analysis in MPC5 cells co-treated with UTI and miR-144-3p. *P < 0.05 and **P < 0.01 versus control group.

Next, our data demonstrated that the expression of miR-144-3p was downregulated by UTI in LPS-treated MPC5 cells (Fig. 3*A*). We predicted the binding site of miR-144-3p and SLC7A11 in a bioinformatic analysis (TargetScan and miRDB, Fig. 3*B*). The mRNA expression along with the luciferase activity of SLC7A11 was inhibited by miR-144-3p mimic in MPC5 cells (Fig. 3*C*, *D*). The protein levels of SLC7A11 were raised by the treatment of UTI while miR-144-3p blocked the protect effect of UTI in MPC5 cells (Fig. 3*E*). Taken together, these data suggest that UTI increased SLC7A11 expression by downregulating miR-144-3p.

### The expression of miR-144-3p blocks the inhibition of UTI on ferroptosis

**Figure 4.**
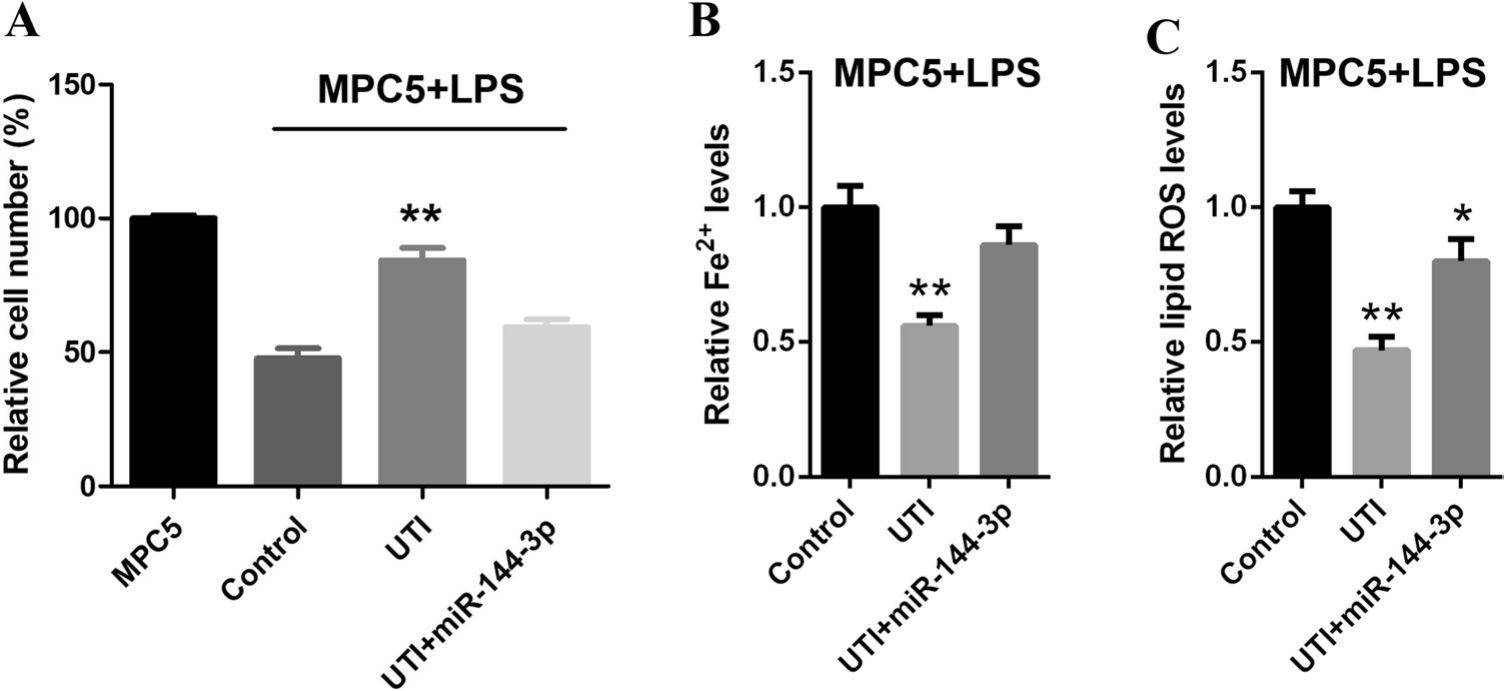
The expression of miR-144-3p blocks the inhibition of UTI on ferroptosis. (*A*) The LPS-stimulated MPC5 cells were co-treated with miR-144-3p and UTI (100 U/mL). The cell viability was detected by MTT assays after 48 h of the treatment. The Fe^2+^ (*B*) and lipid ROS levels (*C*) were detected in LPS-treated podocyte cells with UTI and miR-144-3p. *P < 0.05 and **P < 0.01 versus control group.

Next, we found that the cell viabilities were elevated by UTI in LPS-stimulated MPC5 cells, while miR-144-3p blocked the protective role of UTI (Fig. 4*A*). Moreover, the levels of Fe^2+^ and lipid ROS were declined by the treatment of UTI; in the meantime, the miR-144-3p reversed these expression levels in the LPS-treated podocyte cells (Fig. 4*B*, *C*). Taken together, these data suggest that overexpression of miR-144-3p blocks the inhibition of UTI on LPS-induced ferroptosis.

### UTI alleviates glomerular podocyte injury and proteinuria in in vivo

**Figure 5.**
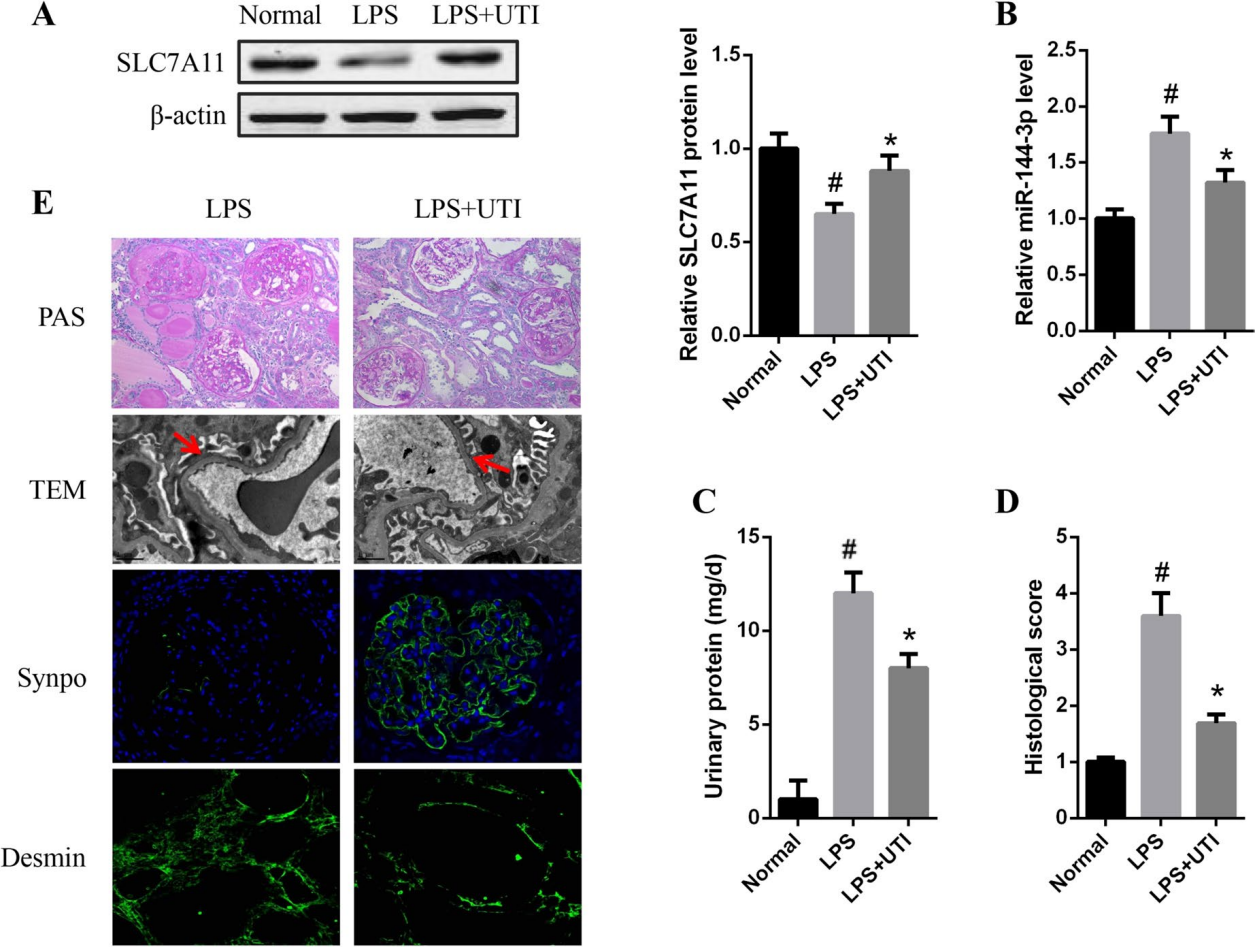
UTI alleviates glomerular podocyte injury and proteinuria in in vivo*. *(*A*) The protein expression of SLC7A11 was detected by western blot analysis. (*B*) The expression of miR-144-3p was analyzed by qPCR assays. (*C*) 24-h urinary protein in three groups of mice. (*D*) Glomerulosclerosis index was semiquantified in 3 groups. (*E*) PAS staining (magnification × 200) and electron microscopy (magnification × 13,500) in LPS and LPS + UTI groups. Immunofluorescence staining showed the expression of synaptopodin and desmin in different groups of mice (magnification × 75). *P < 0.05 versus LPS group; ^#^*P* < 0.05 versus normal group.

As expected, the expression of SLC7A11 was enhanced and miR-144-3p expression was reduced in the renal tissues of UTI-treated mice compared with that in LPS group (Fig. 5*A, B).* As shown in Fig. 5*C*, after UTI treatment, urine protein (24 h) was significantly decreased compared with the LPS group (P < 0.05). The severity of kidney injury was investigated by examination of PAS staining and electron microscopy (Fig. 5*D*, *E*). Matrix expansion and renal fibrosis were remarkably ameliorated in the UTI-treated LPS mice than in those untreated. The electron microscopy exhibited that foot process effacement and GBM thickening were attenuated after receiving UTI treatment. The glomerulosclerosis index was dramatically decreased in the UTI-treated LPS mice than in those untreated (*P* < 0.05; Fig. 5*D*). Immunofluorescence staining showed the expression of synaptopodin was noticeably dropped in the LPS group compared with the control group, while the injury was significantly attenuated after receiving UTI, while an opposite trend was observed for desmin (Fig. 5*E*). Together, these results indicate that UTI attenuates glomerular podocyte injury and proteinuria in in vivo.

## Discussion

Podocyte injury and loss have emerged as a central pathological mechanism underlying glomerular damage and AKI (Kerjaschki 2016). In the present study, as a podocyte toxin, LPS is used to induce experimental renal injury (Chen et al. 1998). We found that compared with the LPS-induced group, UTI treatment significantly ameliorated podocyte injury and prevented podocyte ferroptosis, and ultimately alleviated glomerular fibrosis and kidney injury. The possible mechanisms underlying these effects may have been involved in modulating miR-144-3p and SLC7A11 expression.

High-dose UTI is reported to prevent late-onset acute renal failure after orthotopic liver transplantation. Yang XY *et al*. revealed that UTI ameliorates acute kidney injury induced by crush syndrome inflammation by modulating Th17/Treg cells (Yang et al. 2020). Additionally, UTI was reported to protect against acetaminophen-induced liver injury by alleviating ferroptosis via the SIRT1/Nrf2/HO-1 pathway (Wang et al. 2021). Based on these findings, the present experiment further explored whether UTI ameliorates LPS-induced podocyte injury associated with the inhibition of ferroptosis. In our in vivo and in vitro experiments, we found UTI significantly elevated synaptopodin levels and inhibited desmin expression accompanied by maintaining podocyte cytoskeleton structure.

Ferroptosis, as an emerging programmed cell death, plays critical functions in cancer disease and organ injury. Ferroptosis has been revealed to be important in nephrotoxic folic acid-induced AKI (Martin-Sanchez et al. 2017). XJB-5–131 could inhibit ferroptosis in tubular epithelial cells after I/R injury (Zhao et al. 2020). These reports indicate that targeting ferroptosis may be the potential therapy in kidney injury. Accumulating evidences have suggested that podocytes undergo ferroptosis in progressive kidney disease, which expressed as loss of epithelial markers, such as synaptopodin, together with gaining mesenchymal features (desmin) (Nagata 2016). Synaptopodin is a proline-rich actin-binding protein strongly expressed in highly dynamic cell compartments such as dendritic spines in the brain and podocyte foot processes in the kidney. Originally, we identified synaptopodin as an actin-associated protein of differentiated podocytes, where it is part of the actin-based contractile apparatus in foot processes (FPs). Synaptopodin is a key regulator of podocyte function because bigenic heterozygosity for synaptopodin and CD2-associated protein results in proteinuria and glomerular damage. When podocytes are damaged, the desmin protein (a marker of injury) is affected as an intermediate vector protein. It is conceivable that podocyte ferroptosis results in detachment from the GBM, leading to podocyte dysfunction, proteinuria, and renal injury. Zhang Q *et al*. revealed that Sp1-mediated upregulation of Prdx6 expression prevents podocyte injury in diabetic nephropathy via mitigation of oxidative stress and ferroptosis (Zhang et al. 2021). Notably, non-coding RNA was reported to play a role in podocyte injury in diabetic nephropathy through regulating ferroptosis by targeting miR-188-3p/GPX4 signaling Axis (Jin et al. 2022). This suggests that podocyte depletion might be caused by reduced podocyte adhesion, which is a potential consequence of ferroptosis. In this study, we found podocyte injury through ferroptosis in LPS-induced mice and MPC5 cell line in vitro. LPS decreased podocyte synaptopodin and F-actin expression while increasing desmin expression in vivo and in vitro, which was in agreement with the above studies, suggesting that UTI may inhibit progression of podocyte depletion by inhibition of ferroptosis, providing the valuable evidence of the relationship of UTI and ferroptosis.

miRNAs are posttranscriptional regulators of gene expression, which usually bind to the 3′-UTR of mRNA, leading to inhibition of protein translation or degradation of mRNA. More evidence suggests that specific miRNAs play crucial roles in controlling normal podocyte function (Lin et al. 2014; Nakagawa et al. 2015). Podocyte-specific deletion of dicer markedly causes proteinuria and glomerular damage (Harvey et al. 2008; Nakagawa et al. 2015). In this study, LPS-stimulation was found to effectively increase miR-144-3p expression in the model mice and injured podocyte, while UTI treatment alters this trend, indicating that UTI may be associated with the improvement of podocyte ferroptosis by downregulation of miR-144-3p. In fact, it has been reported that miR-144-3p directly targeted the 3′-UTR of SLC7A11, which represses the transcription of E-cadherin and initiates the process of ferroptosis (Ye et al. 2023). Inhibition of the miR-144-3p was sufficient to reduce ferroptosis in a process requiring upregulation of SLC7A11. To address this, the present study found that miR-144-3p mimic partially blocked UTI-mediated upregulation of SLC7A11 in LPS-exposed differentiated podocytes. These data suggest that UTI may downregulate miR-144-3p expression to alleviate LPS-induced podocyte ferroptosis by enhancing SLC7A11 expression. Therefore, decreasing miR-144-3p expression by UTI may protect against LPS-induced podocyte dysfunction and injury. In an in vivo study, upregulated expression of miR-144-3p and reduced SLC7A11 expression can be seen in the model group. After UTI treatment, LPS-mediated ferroptosis was blocked and then the renal function protected. A new correlation of miR-144-3p/SCL7A11 axis with UTI in the regulation of ferroptosis during podocyte injury in AKI was indicated in the study.

It should be noted that there are some limitations of this study. miR-144-3p/SLC7A11 axis may be just one pathway underlying the protective role of UTI in preventing AKI progression. Thus, the transcriptional cascade of UTI with its downstream transcription factors is important to regulate branched pathways downstream of AKI, and more in-depth investigations are needed in the future. On the other hand, ferroptosis, apoptosis, and other forms of cell death may have crosstalk between each other, and studies are needed to explore this in future investigations.

## Conclusions

Our study demonstrated that UTI ameliorated LPS-induced ferroptosis and subsequent podocyte dysfunction through decreasing miR-144-3p expression. These findings might provide a potential novel therapeutic option for AKI and other renal diseases affecting podocytes.
